# A Systematic Review of Case Reports of a Rare Dermatological Condition: Elastosis Perforans Serpiginosa

**DOI:** 10.7759/cureus.40296

**Published:** 2023-06-12

**Authors:** Smruti M Besekar, Sangita D Jogdand, Waqar M Naqvi

**Affiliations:** 1 Pharmacology, Jawaharlal Nehru Medical College, Datta Meghe Institute of Higher Education and Research, Wardha, IND; 2 Research, Humen Edutech, Nagpur, IND; 3 Pharmacology and Therapeutics, Jawaharlal Nehru Medical College, Datta Meghe Institute of Higher Education and Research, Wardha, IND; 4 Physiotherapy, Gulf Medical University, Ajman, ARE

**Keywords:** wilson disease, ehlers-danlos syndrome, penicillamine, case reports, elastosis perforans serpiginosa

## Abstract

A systematic review was carried out on a rare dermatological condition affecting papillary dermal tissue fibers of the skin known as elastosis perforans serpiginosa (EPS). The aim of this review was to highlight this skin disease, its association with other medical conditions, and its management. The search was conducted by using the keywords "elastosis perforans serpiginosa" and "case reports" in the databases. A total of 10 case reports were analyzed and presented by the parameters like age, gender, chief complaints, and medical history. The most common causes of EPS were drug-induced and occurred along with the Wilson disease. The study concluded that as EPS is an uncommon disease with few instances, there is a need for further research to analyze randomized controlled trials that have been conducted in relation to the condition.

## Introduction and background

Elastosis perforans serpiginosa (EPS) has been clinically recognized since 1953. Removal of papillary dermal elastic fibers through skin papules is a defining feature of the disorder. Hyperkeratotic red- or skin-colored papules grouped in circular patterns are typical EPS appearances that can be asymptomatic. The most common sites of these lesions are the upper extremities, the face, the neck, and the lower limbs. This illness is more prevalent in men, mostly in young adults [1].

The disorder has been classified as the initial type, which is congenital, and the other form is related to intrinsic illnesses such as cutis laxa, pseudoxanthoma elasticum, Down syndrome, osteogenesis imperfecta, Ehlers-Danlos syndrome (EDS), and Marfan syndrome. The last form is drug-induced, such as D-penicillamine [2-4]. A systematic review of EPS case reports was conducted to highlight this rare dermatological condition and its management.

## Review

Research methodology

**Figure 1:**
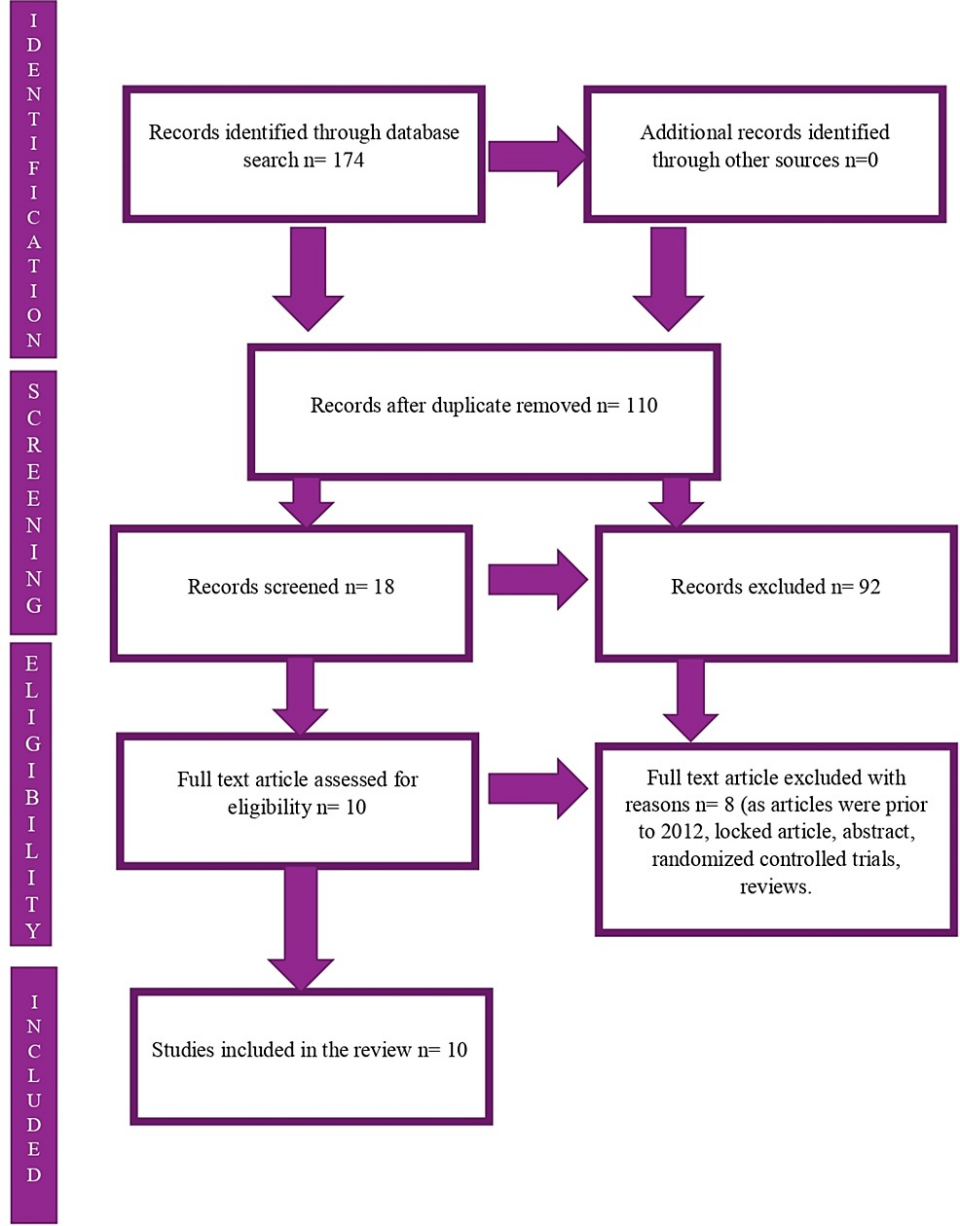
Search strategy for elastosis perforans serpiginosa from the databases

This systematic review was performed according to the updated 2020 Preferred Reporting Items for Systematic Reviews and Meta-Analyses literature search extension (PRISMA-S) statement [5]. PubMed, Cochrane Library, PsycINFO, and Scopus databases were searched for case reports, case series, and letters to the editor published between 2012 and 2022, and also discovered local and international case presentations. PubMed had 173 articles and Scopus had one relevant article. The search terms were combined to explore the databases using the Boolean operator "AND" in between the text terms "elastosis perforans serpiginosa" and "case reports." The papers included in this review covered the years 2012-2022 and were written in English, completely free full-text articles, and represented cases and clinical findings, together with signs and symptoms, diagnosis, and treatment. Peer-reviewed publications, observational studies, and randomized controlled trials conducted before 2012, those without full text, and texts with titles that did not include elastosis perforans serpiginosa were eliminated. Finally, only the PubMed database was considered for the analysis, because it qualifies strict inclusion and exclusion norms. The inclusion and exclusion criteria for the case reports are shown in Figure 1.

Results

From the 174 articles, only 10 case reports were found that included the term “elastosis perforans serpiginosa” and “case report” after using the inclusion criteria. These case reports included the majority of cases with D-penicillamine-induced EPS, EPS with other conditions such as Wilson disease (WD; five cases), Ehlers-Danlos syndrome (one case), and pseudoxanthoma elasticum (two cases), and the treatment management of EPS, respectively. Table 1 included the data about the authors, journal, date of publication, patient’s age, gender, chief complaint, and associated medical history.

**Table 1:** Information on the study’s author, gender, age, and associated medical/past history

Sr. No.	Author	Journal name	Year	Age/gender	Chief complaint	Associated medical/past medical history
1	Heusinkveld et al. [6]	International Journal of Women's Dermatology	2021	53 years/female	Two painful 2-cm nodules at the apices of an excision scar	Squamous cell carcinoma excision before two years, recurrent cystinuria nephrolithiasis after intake of penicillamine for 43 years, troponin for one year
2	Lee et al. [7]	Annals of Dermatology	2014	35 years/male	Papules of hyperkeratosis on his anterior neck	No associated medical history
3	Rinaldi et al. [8]	Case Reports in Dermatological Medicine	2019	15 years/male	On the left and right sides of his posterior neck, he exhibited two isolated patches of erythematous umbilicated papules grouped in a serpiginous pattern, as well as extensive umbilicated papules that had been present for more than two years	No associated medical history
4	Samal et al. [9]	Chinese Medical Journal	2020	34 years/male	For two years, the patient complained about rash without any itching and discomfort	Hepatolenticular degeneration since 20 years and a history of intake of penicillamine
5	Campanati et al. [10]	Acta Dermatovenerol Alp Pannonica Adriat	2014	40 years/female	For one year, the nape of the neck has had inflammatory lesions	No associated medical history
6	Chisti et al. [11]	Annals of Saudi Medicine	2019	37 years/male	Itchy skin lesions for three months	Wilson disease and on D-penicillamine for 22 years
7	Venkatachalam et al. [3]	Indian Dermatology Online Journal	2016	22 years/female	Appeared with a three-year history of excessive neck skin folds and a six-month history of an asymptomatic neck eruption	First-degree consanguinity
8	Menzies et al. [12]	BMJ Case Reports	2015	42 years/female	Several growing skin lesions since five years	Wilson disease and were on penicillamine for 20 years
9	Pallesen et al. [13]	Dermatology Online Journal	2019	26 years/female	The recent development of skin lesions	Ehlers-Danlos syndrome
10	Liang et al. [14]	Indian Journal of Dermatology, Venereology and Leprology	2016	(1) 32 years/male; (2) 38 years/male	(1) With a two-year history of annular, serpiginous, geographic, and other atypical patterns of 3-5 mm in diameter, red, somewhat irritating keratotic papules on the neck. (2) On the neck, with central clearing, atrophy, and hypopigmentation, as well as somewhat irritating, garnet-colored papules that coalesce into serpiginous plaques with elevated hyperkeratotic rims	Both were cases of Wilson disease and managed by D-penicillamine

Discussion

In this systematic review, EPS was found to be associated with medical conditions such as WD, pseudoxanthoma elasticum, and Ehlers-Danlos syndrome, and it is also a consequence of drug reactions [6,9,11,14,15].

Based on the clinical and histological results, EPS was diagnosed. Histopathological analysis of EPS revealed localized or perforated epidermal hyperplasia, eosinophilic deteriorated elastic fibers, and a thin vertical transepidermal channel that included nuclear debris, granular basophilic debris, scant mononuclear inflammatory infiltrate, and bumble brush appearance of elastic fibers [3,6-10,12-15]. Histological evaluation of EPS specimens is usually performed with Verhoeff-Van Gieson staining, Gomori methenamine silver staining, and periodic acid-Schiff with diastase [1].

The most common management for good prognosis of EPS is calcipotriene ointment, topical tretinoin, oral isotretinoin glycolic or salicylic acid, topical tazarotene intralesional and topical corticosteroid curettage cryotherapy narrow band ultraviolet B radiation, and erbium-doped yttrium aluminum garnet (Er:YAG), carbon-dioxide (CO2), and dye lasers [1]. Moreover, laser procedures have excellent safety and efficacy records. Face redness caused by dilated or extra blood vessels can be safely and successfully treated using pulsed dye lasers that use heat-transfer light and a solution containing an organic dye to produce a laser effect, they are known as "pulsed-dye" lasers [16]. This targets specific parts of the skin with delicate, yet powerful, light bursts that cause the destruction of damaged blood vessels due to heat, and the skin around them is protected. The case showed the beneficial effect of pulse dye laser therapy as the patient received eight treatment sessions over a period of four years and received positive results of 75% clearance of the erythematous itchy lesions, and no side effects have been reported. After 15 years of therapy, no recurrence of lesions was noted [8,16].

Another case showed a patient who was initially treated with topical corticosteroids and oral isotretinoin, and because of the failure of this therapy, the patient was shifted to intralesional steroids and topical allium cepa-allantoin-pentaglycan gel, which showed a beneficial effect [10]. Since isotretinoin received FDA approval in 1982, it has had a significant impact on dermatology [17]. In particular, it has transformed the treatment of severe nodular-cystic acne, miraculously curing the condition [18]. However, isotretinoin has been used "off-label" to treat more than 50 dermatological disorders, other than acne, because of its potent anti-inflammatory, immunomodulatory, antineoplastic, and other pharmacological properties. It is crucial to ensure that it is properly used as its implementation spreads. While certain "off-label" treatments have been demonstrated to be helpful in well-researched trials, others are still in use, despite recent studies demonstrating the ineffectiveness of isotretinoin. It has been used for the management of rosacea, psoriasis, pityriasis rubra pilaris, condyloma acuminatum, granuloma annulare, Darier’s disease, systemic cutaneous lupus erythematosus, non-melanoma skin cancer, and hidradenitis suppurativa [19]. The use of topical allium cepa-allantoin-pentaglycan gel was also found to be beneficial in reducing neoangiogenesis in 15 patients with hypertrophic scars and keloids, resulting in the clinical improvement of skin lesions [20].

EPS is associated with WD, which is characterized by increased copper accumulation in the liver and basal ganglia. It is a hallmark of the autosomal recessive condition, with hepatic and neurological consequences, and is treated with two chelating drugs, penicillamine and trientine (Syprine), which remove copper from the body. Trientine is less likely to have adverse effects than penicillamine [21]. Fever, rash, and kidney and bone marrow issues are possible side effects of penicillamine. Cuprimine is a generic name for penicillamine and is used for the treatment of WD, rheumatoid arthritis, arsenic, and lead poisoning [21,22]. Penicillamine (D-b,-dimethyl cysteine) was first identified as a penicillin breakdown product by Abraham et al. in 1943 [23]. Hyperkeratotic papular lesions, commonly arranged in an annular or serpiginous form on any body surface, including the lips, oral mucosa, and glans penis, have been described as D-penicillamine (DPA)-induced EPS. These lesions cannot be distinguished from idiopathic EPS lesions [24].

EPS with a history of Wilson's disease was found to have distinctive Kayser-Fleischer (KF) rings, which are common ophthalmologic findings due to the buildup of silver, and are caused by excessive copper deposition on the inner surface of the cornea in the Descemet membrane, which is diagnosed with slit light, especially in the early stages, unless there is severe copper overload, and the rings may be seen without a microscope. Although they disappear with treatment and recur as the disease progresses, KF rings do not impede vision. Wilson’s disease is not the only chronic cholestatic disorder in which KF rings are present; children with primary biliary cholangitis and other chronic cholestatic disorders also exhibit it [25,26]. In the cited case, the patient showed characteristic KF rings and skin biopsy revealed distinct acanthotic cutis with broad, rough elastic fibers, serrations, and buds sprouting perpendicular to the elastic fibers. This led to the conclusion that D-penicillamine should be stopped, but stopping the medication was not an option; therefore, topical application of tazarotene gel was advised [14]. After a confirmed diagnosis of EPS infection, another patient with the same symptoms, except urine copper level, was within the normal range and was treated with topical corticosteroids [11].

Less than 20 cases of EPS associated with WD treated with DPA, characterized by histopathologically verified skin lesions, have been documented in the literature. Male patients were more likely to have EPS, which is typically present in the neck, axilla, and arms; only three instances included the glans penis. A minimum of five years and a maximum of 29 years are required for DPA therapy to start working on the skin lesion [27].

Dermal cross-linking of collagen and elastic fibers by the copper-dependent enzyme lysyl oxidase is the initial step in the D-penicillamine-induced EPS process. D-penicillamine chelates copper cofactor, which obstructs lysyl oxidase activity and results in the accumulation of aberrant elastic fibers. Second, D-penicillamine can reduce collagen cross-linking by preventing collagen production after translation. Similarly, another case report found that biopsy revealed multinucleated histiocytic giant cell inflammatory reactions and short, fragmented eosinophilic fibers in the dermis that were focally aggregating [24].

In this case report, trientine was administered to the patient in an effort to stop penicillamine, which caused Wilson's disease symptoms to worsen, requiring the re-administration of penicillamine; hence, creams containing copper and a lower dose of penicillamine were used to treat the patient's skin [28]. New treatments have been introduced for Wilson's disease, including gene therapy, bis-choline tetrathiomolybdate, zinc salts, methanobactin, and trientine [19-21].

Pseudoxanthoma elasticum (PXE) is a chronic condition marked by mineralization and the buildup of deposits of calcium and other minerals in elastic fibers. Elastic fibers can be impacted by mineralization in PXE in the skin, eyes, and blood vessels, as well as less frequently in other regions, such as the digestive system. Papules, yellowish pimples that appear as joint bends, are sometimes present on the necks, underarms, and other skin-touching areas of people with PXE (flexor areas). It also causes abnormal pigmentation in the retina, known as peau d'orange. As such, there were no cure, lifestyle modification, or dietary restrictions [29]. In the case associated with pseudo-pseudoxanthoma elasticum and treatment with acitretin, the second generation of retinoid used for the management of psoriasis and has other negative effects such as cheilitis and alopecia, the patient showed improvements at the low dose and showed flare on discontinuing the penicillamine treatment [15].

With varying degrees of success, various therapeutic techniques, such as cryotherapy, electrocautery, photodynamic therapy, imiquimod, tazarotene gel, intralesional steroids, curettage, and laser, have been used to treat EPS caused by penicillamine. However, there is no medical intervention for pseudo-PXE to accelerate the healing of skin lesions and restore elasticity [30,31].

The clinical symptoms of Ehlers-Danlos syndrome include a collection of genetic connective tissue disorders involving brittle blood vessels, atrophic scarring, hypermobile joints, and hyperelastic skin. Although determining the gene encoding collagen or the interacting proteins is required to determine the type of EDS, it is primarily diagnosed clinically [32-34]. The management included preventing the risk of complications, physical therapy for joint mobility, the use of sunscreen and mild soaps for skin protection, and supplements of vitamin C for bruise reduction [15].

This was the first literature analysis conducted on the rare dermatological condition EPS and its case reports, which highlighted its symptoms, findings, and associated medical conditions with useful data on the management and therapeutic approach. The majority of the case reports in this systematic analysis were drug-induced EPS and had WD-related medical histories. Therefore, it is important to determine the appropriate treatment for this condition in the future research.

The drawback of this review is that it included only a small number of cases and patients; hence, the extent of the disease cannot be understood. An equal number of males and females showed EPS conditions; therefore, gender predominance cannot be distinguished. As it is a rare condition and literature on this topic is scarce, hence there is a less number of studies to conclude a better treatment.

## Conclusions

The study concluded that long-term administration of DPA would result in EPS conditions; hence, physicians must be alert about the negative effects of long-term penicillamine prescriptions. Discontinuation of DPA does not ensure that the skin condition will not continue to worsen. Close monitoring is needed to avoid lethal systemic conditions. Alternatives such as pulse dye therapy, acitretin, and steroids have shown good effects on patients. As it is a rare condition, less number of cases were reported to date. More awareness is needed to disperse the knowledge about EPS for the physician. Furthermore, future studies based on randomized controlled trials are needed to understand the new treatment of EPS.
